# *De-novo* Assembly of *Limnospira fusiformis* Using Ultra-Long Reads

**DOI:** 10.3389/fmicb.2021.657995

**Published:** 2021-04-16

**Authors:** McKenna Hicks, Thuy-Khanh Tran-Dao, Logan Mulroney, David L. Bernick

**Affiliations:** ^1^Department of Microbiology and Environmental Toxicology, University of California, Santa Cruz, Santa Cruz, CA United States; ^2^Department of Biomolecular Engineering, University of California, Santa Cruz, Santa Cruz, CA United States

**Keywords:** *Limnospira*, *Arthrospira*, *Spirulina*, genome, nanopore, axenicity, cyanobacteria, long-read

## Abstract

The *Limnospira* genus is a recently established clade that is economically important due to its worldwide use in biotechnology and agriculture. This genus includes organisms that were reclassified from *Arthrospira*, which are commercially marketed as “*Spirulina*.” *Limnospira* are photoautotrophic organisms that are widely used for research in nutrition, medicine, bioremediation, and biomanufacturing. Despite its widespread use, there is no closed genome for the *Limnospira* genus, and no reference genome for the type strain, *Limnospira fusiformis*. In this work, the *L. fusiformis* genome was sequenced using Oxford Nanopore Technologies MinION and assembled using only ultra-long reads (>35 kb). This assembly was polished with Illumina MiSeq reads sourced from an axenic *L. fusiformis* culture; axenicity was verified via microscopy and rDNA analysis. Ultra-long read sequencing resulted in a 6.42 Mb closed genome assembled as a single contig with no plasmid. Phylogenetic analysis placed *L. fusiformis* in the *Limnospira* clade; some *Arthrospira* were also placed in this clade, suggesting a misclassification of these strains. This work provides a fully closed and accurate reference genome for the economically important type strain, *L. fusiformis*. We also present a rapid axenicity method to isolate *L. fusiformis*. These contributions enable future biotechnological development of *L. fusiformis* by way of genetic engineering.

## Introduction

*Limnospira* is a recently established genus that contains three species reclassified from *Arthrospir*a based on morphological, molecular, and ecological differences between the two genera (Nowicka-Krawczyk et al., 2019). *Spirulina (Arthrospira) platensis* SAG 85.79 (SAG 85.79 = CCALA 026 = UTEX 2340), *Arthrospira maxima* UTEX 2720, and *Arthrospira platensis* PCC 8005 were reclassified to *Limnospira fusiformis*, *L. maxima*, and *L. indica*, respectively (Voronichin, 1934; Nowicka-Krawczyk et al., 2019). *Limnospira fusiformis* is the type species of this new genus (Nowicka-Krawczyk et al., 2019). Although more strains possibly fall under the new *Limnospira* genus, there is insufficient evidence to reclassify them (Nowicka-Krawczyk et al., 2019). Both *Arthrospira* and *Limnospira* comprise photosynthetic filamentous cyanobacteria that form multicellular cylindrical trichomes (Belay and Gershwin, 2007; Nowicka-Krawczyk et al., 2019). Their cultivation is simple, inexpensive, and their alkaline growth preference makes commercial production less prone to living contaminants (Kebede and Ahlgren, 1996; Nowicka-Krawczyk et al., 2019). These aspects make *Limnospira/Arthrospira* attractive for use in many biotechnological and agricultural applications, including wastewater treatment, biofuels, biomanufacturing, medicine, and nutritional supplementation (Belay et al., 1993; Tokuşoglu and üUnal, 2003; Belay and Gershwin, 2007; Abed et al., 2016; Jiang et al., 2017; Aikawa et al., 2018; Czerwonka et al., 2018; Mehan et al., 2018; Zinicovscaia et al., 2018). Both *L. fusiformis* and *L. maxima* are mass-produced, economically important, and are approved by the Food and Drug Administration (FDA) for human consumption (Belay and Gershwin, 2007; Sili et al., 2012; Nowicka-Krawczyk et al., 2019). Both are, confusingly, known as “Spirulina” in commercial settings (Sili et al., 2012; Nowicka-Krawczyk et al., 2019).

Documented consumption of “Spirulina” dates to ancient Aztec civilization (Walker et al., 2014; Wan et al., 2016). Presently, “Spirulina” is globally consumed as a nutritional supplement because they are extremely nutrient dense (Campanella et al., 1999; Tokuşoglu and üUnal, 2003; Babadzhanov et al., 2004). A recent market research report predicted the global “Spirulina” market to reach $629.6 million and 68,025.2 tons by 2025 (Spirulina Market - Global Opportunity Analysis and Industry Forecast, 2019–2025).

In addition to being a massively utilized commercial “Spirulina” strain, *L. fusiformis* is one of two that have a publicly available genetic system (*A. platensis* C1 being the second) (Jeamton et al., 2017; Dehghani et al., 2018). Despite its economic importance and status as the type species for the *Limnospira* genus, a published reference genome for *L. fusiformis* does not yet exist.

There are many sequenced genomes in the “Spirulina” grouping; many of these were sequenced using short-read technologies resulting in multi-contig assemblages due to their repeat ridden character (Fujisawa et al., 2010; Carrieri et al., 2011; Cheevadhanarak et al., 2012; Sili et al., 2012; Lefort et al., 2014; Dong et al., 2015; Xu et al., 2016; Suzuki et al., 2019). A recent *L. fusiformis* KN assembly produced a non-final 5.78 Mb genome composed of 401 scaffolds with an N50 of 32,852 (GCA_014489865.1), demonstrating the limitations of a short-read approach. The nearest relative with an associated genome publication in the *Limnospira* clade is of *L. indica* PCC 8005 (formerly *A. platensis* PCC 8005, or *A. nitrilum* PCC 8005), and like other members of this group, Janssen et al. found its genome to be highly repetitive with components such as CRISPR arrays and transposable elements (Janssen et al., 2010). Repeat-rich genomes are difficult to close, and an unclosed genome can lead to missing genes and other assembly errors. Furthermore, an unclosed genome makes it difficult to identify contaminating sequences, which can confuse downstream genomics studies. Fully closed genomes provide a complete and accurate picture of an organism’s genetics, providing useful biological insights. Long-read sequencing can accurately span repetitive regions, making it a valuable approach for closing repeat rich genomes (Fraser et al., 2002; Amarasinghe et al., 2020). Indeed, long-read sequencing technology has produced the only closed genomes in the “Spirulina” grouping (*Arthrospira* sp. TJSD092 (GCA_003060805.1) and *“Arthrospira* sp.*”* PCC 9108 (GCA_016745315.1).

In this study we sequenced, assembled, and closed the 6.42 Mb genome in a single contig using Oxford Nanopore Technologies (ONT) MinION long-read technology, and polished the assembly with a high-accuracy, short-read Illumina MiSeq library sourced from an axenic culture. With this work we present a novel axenicity protocol and a fully closed genome for *L. fusiformis*, the type strain for the newly described *Limnospira genus*.

## Materials and Methods

### Bacterial Strains and Culture Conditions

A xenic culture of *Limnospira fusiformis* was purchased from the University of Texas Culture Collection of Algae (UTEX 2340). The cells were grown in modified SAG medium (162 mM NaHCO_3_, 38 mM Na_2_CO_3_, 2.9 mM K_2_HPO_4,_ 29.4 mM NaNO_3_, 5.74 mM K_2_SO_4_, 17.1 mM NaCl, 0.81 mM MgSO_4_ ⋅ 7H_2_O, 0.27 mM CaCl_2_ ⋅ 2H_2_O, 12.13 μM Na_2_EDTA ⋅ 2H_2_O, 2.16 μM FeCl_3_ ⋅ 6H_2_O, 1.32 μM MnCl_2_ ⋅ 4H_2_O, 0.22 μM ZnCl_2_, 0.134 μM CoCl_2_ ⋅ 6H_2_O, 0.154 μM Na_2_MoO_4_ ⋅ 2H_2_O, 0.08 μM CuSO_4_ ⋅ 5H_2_O, 0.15 μM ZnSO_4_ ⋅ 7H_2_O, 10 μM H_3_BO_3_, 0.1 μM cyanocobalamin) shaking at 150 rpm in 30°C with 12 h 80 μmol photons per m^2^s light cycles using a 90 CRI light source with a color temperature of 3,000 K.

### Optimum Centrifugal Force for Selective Gas Vesicle Collapse

*L. fusiformis* gas vesicle persistence was tested using various centrifugal forces. A *L. fusiformis* culture in log-phase settled overnight and buoyant cells were collected from the surface layer. The cells were centrifuged for 10 min at 2,000, 4,000, 8,000, or 16,000 g. As a control, one sample was not centrifuged. Both the pellet and surface cells from each sample were microscopically examined for changes in gas vesicles via phase-contrast microscopy at 600× magnification using a Nikon Eclipse E400.

### Physical *L. fusiformis* Purification Treatments

Xenic *L. fusiformis* cultures were inoculated (OD_750_:0.2) in modified SAG medium. Culture growth was measured in 24 h intervals via OD_750_. Once the cultures reached the log phase of growth, they were incubated without shaking overnight to allow the formation of a layer of floating cells. Floating filaments were collected from the culture’s liquid surface and vortexed at medium speed for 15 s followed by 15 s on ice for 1 min. The vortexed culture was filtered using a 40 μm nylon cell strainer and washed with sterile SAG media to remove contaminants smaller than 40 μm. Filaments that remained on the strainer were suspended in 1 mL of sterile SAG, briefly vortexed, then centrifuged for 10 min at 8,000 g to separate the cellular mass into two phases: filaments pelleted with contaminants and buoyant filaments at the surface. The surface layer of filaments were collected, resuspended in sterile SAG, and centrifuged at 8,000 g a second time. The final surface layer was examined using dark-field microscopy using a Nikon Eclipse E400 at 200× magnification to assess contaminants. A portion of these cells had DNA extracted for Nanopore sequencing and genome assembly. The remaining cells were treated with further chemical purification to establish an axenic culture.

### Chemical *L. fusiformis* Purification Treatments

Sterile SAG media was supplemented with 65 μg/mL ampicillin, 77 μg/mL cefoxitin, and 100 μg/mL meropenem (Choi et al., 2008; Sena et al., 2011). The pH of the media was adjusted to 12.15 using NaOH, then re-sterilized by 0.22 μm filtration. Cells that were physically treated were inoculated into this media and incubated in the dark for 4 days at 150 rpm and 30°C.

After 4 days, the entire culture was filtered through a 1 μm polycarbonate filter. Cells retained on the membrane were gathered and suspended in 1 mL sterile SAG medium. The cell suspension was centrifuged and the surface layer was collected. These surface layer cells were examined for contaminating species using dark-field and phase-contrast microscopy using a Nikon Eclipse E400 at 200× and 600× magnification. DNA was extracted from these cells and sequenced with Illumina sequencing to polish the genome assembly and to verify axenicity.

### DNA Extraction

We adapted a CTAB-based *A. platensis* DNA extraction method to purify and preserve high molecular weight DNA (Morin et al., 2010). Throughout the method, to preserve high molecular weight DNA we used wide-bore tips, pipetted slowly, and only mixed with gentle inversion and finger-flicking to prevent mechanical DNA shearing. *L. fusiformis* cells were resuspended in 0.5 mL sterile extraction buffer (0.15 M NaCl, 0.1 M EDTA, pH 8.0) and subjected to three freeze-thaw cycles using dry ice and a 37°C bath to damage the cell walls and increase the efficiency of cell lysis. The cells were centrifuged for 10 min at 8,000 g, collected, and resuspended in CTAB buffer (75 mM Tris-HCl, 2% CTAB, 1.4 M NaCl, 1 mM EDTA, H_2_O, pH 8). These cells were enzymatically lysed with 50 mg of lysozyme at 37°C for 30 min. The lysed cells were incubated at 37°C for 1 h with 2% SDS, 5 mg/mL proteinase K, and 100 μg/mL RNase A. Following this incubation, the lysed cells were gently mixed by slow inversion and incubated at 65°C for 10 min to optimize the formation of CTAB -protein and -polysaccharide complexes.

The sample was incubated with 1 volume of 24:1 chloroform: isoamyl alcohol on ice for 30 min. The sample was centrifuged for 10 min at 3,500 g and the aqueous phase was transferred to a fresh tube. One volume of phenol:chloroform:isoamyl alcohol (25:24:1) was added, mixed with gentle inversion, and centrifuged for 3 min at 3,500 g. The aqueous phase was transferred to a fresh tube and the phenol:chloroform:isoamyl wash was repeated until the interphase was cleared of flocculent material. The aqueous phase was gently washed with one volume of 24:1 chloroform:isoamyl alcohol, centrifuged for 3 min at 3,500 g, and transferred to a fresh tube. The chloroform:isoamyl alcohol wash was repeated 4 more times.

The final aqueous phase was gently mixed with 1/10 volume 3 M NaOAc (pH 5.4) and 2.5 volumes of 100% ethanol to precipitate DNA. The sample was incubated at −20°C overnight, then centrifuged at 3,500 g for 1 h at 4°C to pellet the DNA. The supernatant was removed, and the DNA was washed twice with 70% ethanol and mixed by inversion. Following the last wash, the DNA pellets were allowed to air dry. TE (1X) buffer was added to the DNA pellet and it was then incubated at 37°C until fully dissolved. The DNA purity was assessed with a Nanodrop UV/VIS spectrophotometer and the size was observed on a 0.5% agarose gel alongside a NEB 1 kb extended ladder (#N3239S). We consistently extracted pure high molecular weight DNA using this method (Supplementary Figures 1, 2). High molecular weight DNA was selected for Nanopore sequencing via gel extraction.

### DNA Sequencing

*L. fusiformis* cells that had undergone only physical purification were sequenced using one Oxford Nanopore Technologies’ (ONT) MinION flowcell. The DNA was prepared for sequencing using the SQK-LSK109 protocol following the manufacturer’s instructions. DNA extracted from the axenic *L. fusiformis* culture—which had undergone both physical and chemical treatments—was shipped to the University of California Davis DNA Technologies and Expression Analysis Cores for Illumina library preparation and sequencing. The DNA was sheared and size selected for 500 bp fragments and sequenced using a single Illumina MiSeq 2 × 300 run using index AAGGTACA, which accounted for 17% of the 21.8 M reads with an overall Q30 > 80%.

### Long-Read Genome Assembly

Default settings were used for all tools unless otherwise specified. The MinION reads were basecalled using ONT’s basecaller, Bonito v0.1.5^1^. The basecalled MinION reads were filtered to include reads > 35 kb and these were assembled using Shasta v0.4.0 (Shafin et al., 2019). This assembly was polished using PEPPER v0.1.1^2^, followed by three rounds of polishing with the Illumina data using Pilon v1.22 (Walker et al., 2014). NCBI’s Prokaryotic Genome Annotation Pipeline was used to annotate the genome^3^. BUSCO v4.1.4 was used to assess genome quality by tabulating the 773 single-copy core cyanobacterial orthologs represented in the cyanobacteria_odb10 model (Seppey et al., 2019). NCBI tblastn and blastp (Altschul et al., 1990) were used to identify missing orthologs reported by BUSCO. The genome was oriented using *dnaA* as a marker for the origin of replication; it was placed on the top strand and as the first gene using Geneious v11.0.5. Repeats were identified using the Repeat Finder v1.0.1 Geneious plugin. PlasmidSPAdes v3.12.0 and Bandage v0.8.1 were used to identify potential plasmids within the Illumina data (Wick et al., 2015; Antipov et al., 2016). PhyML v2.2.4 with 1,000 bootstrap steps and a Jukes-Cantor genetic distance model was used to build a maximum-likelihood phylogenetic tree using published *cpcAB* gene sequences (Guindon et al., 2010). The phylogenetic tree was visualized with SeaView v5.0.4 (Gouy et al., 2010).

### Short-Read Genome Assembly

**FIGURE 1:**
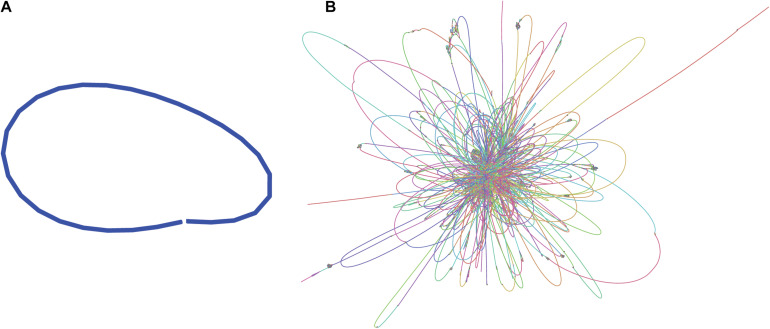
Assembly graphs of *L. fusiformis* assemblies using **(A)** nanopore data (Shasta), **(B)** illumina data (SPAdes) (images generated using Bandage: Wick et al., 2015).

Illumina paired-end sequences were merged and adapters were trimmed using SeqPrep^4^, specifying a minimum overlap of 50 bases and enabling merging with the -s option. Of the 3.7 M original read-pairs, 2/3 were able to be merged, resulting in 2.36 M merged reads and 1.21 M unmerged read-pairs. The 1.2 M read-pairs and 2.36 M merged reads were assembled with the SPAdes-bwa mem v3.12.0 assembler using parameters -t 16 (16 threads) -v 1 (verbose level errors). This assembly was used only for visualization purposes (Figure 1B).

### Axenicity Verification

Axenicity was verified using RNAmmer 1.2 to detect ribosomal RNA genes in axenic Illumina contigs (Lagesen et al., 2007). Organisms were identified by their ribosomal RNA genes using NCBI BLAST (Altschul et al., 1990).

## Results

### Genome

#### Sequencing Approach Produced a Closed Genome Assembly

**FIGURE 2:**
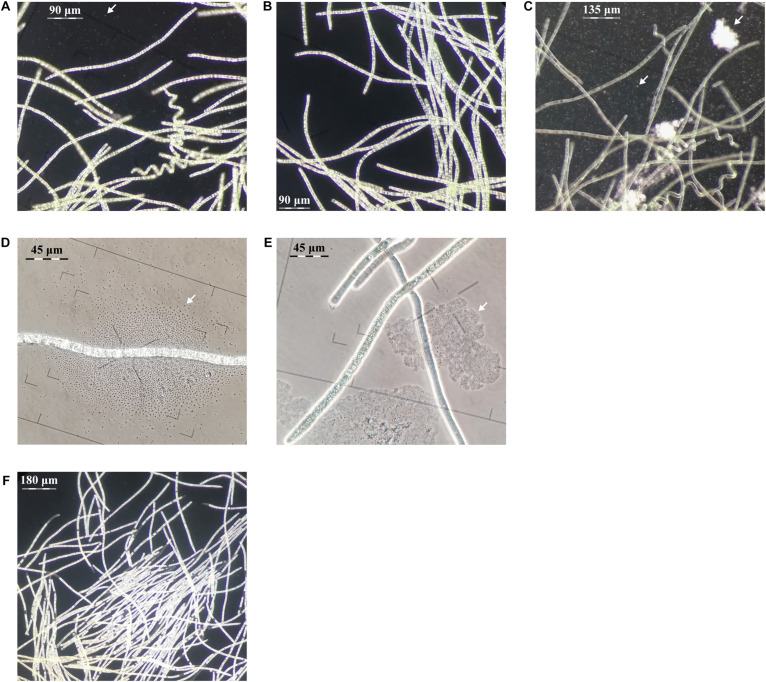
Evaluation of centrifugation, elevated pH and antibiotic purification methods for *L. fusiforms* (contaminants shown with arrows). **(A)** Buoyant cells obtained from a culture of *L. fusiformis* before any treatment that contained contaminants. Applying only centrifugation resulted in **(B)** a surface layer of partially purified trichomes and **(C)** a pellet containing debris and contaminants. **(D)** Buoyant cells obtained from a culture of *L. fusiformis* before any treatment. **(E)** Applying only elevated pH (12.15 pH for 4 days) resulted in a culture with lysed contaminants (likely *Microcystis* sp. based on morphology; Sant’Anna et al., 2008). **(F)** The combination of filtration, centrifugation **(B)**, pH treatments **(E)**, and an antibiotic cocktail resulted in an axenic *L. fusiformis* culture.

The *L. fusiformis* (Table 1) genome is highly repetitive, which may cause misassembly with short-read data, such as that produced by Illumina sequencing. We used long-read nanopore sequencing to assemble the genome to circumvent issues posed by repeated sequences. We performed nanopore sequencing on DNA that was extracted from a physically treated culture that resulted in predominantly *L. fusiformis* DNA (Figure 2B and Supplementary Figure 13). Ultra-long read libraries maximize the overlaps between reads, minimize the opportunities for misassembles caused by common repeats, and thereby reduce the need for a completely axenic culture. We acquired 1.72 M reads from a single MinION flowcell and filtered for reads > 35 kb (17,345 reads). The filtered reads resulted in a fully closed genome assembly with 114× coverage, at a predicted base accuracy of ∼96.4% (Jain et al., 2018; Mulroney et al., 2020). To ensure the most reliable genome assembly, we isolated and sequenced an axenic culture using selective gas vesicle collapse, filtration, antibiotic treatments and alkaline pH selection. DNA extracted from this culture was sequenced using short-read Illumina (157× coverage). The nanopore genome assembly was polished using this short-read library, with a predicted > 99% average base accuracy (Jain et al., 2018) (see section “Materials and Methods”). This produced an NCBI-classified *complete* assembly level genome (Kitts et al., 2016) with an N50 of 6.42 Mb. In contrast, the short-read only assembly had an N50 of 51.6 kb, thus it was discarded and not used outside of Figure 1B. The advantage of the ultra-long read library is clearly apparent when comparing assemblies using the two techniques independently (Figure 1). When combined, we produced a closed 6.42 Mb circular genome assembled as a single chromosome (Table 2).

**TABLE 1:** Classification and general features of *L. fusiformis* according to the MIGS standard (Field et al., 2008).

Classification and general features of *Limnospira fusiformis* SAG 85.79		
Property	Term	References
Current classification	Domain *Bacteria* Phylum *Cyanobacteria* Class *Cyanophyceae* Order *Oscillatoriales* Genus *Limnospira* Species *fusiformis SAG 85.79*	TAS^a^ TAS^b^ NAS TAS^a^ TAS^b^
Cell shape	Spiral	NAS
Sporulation	None	NAS
Encoded traits	Antibiotic resistance *beta-lactam and fluoroquinolones*	IDA
Temperature range	20–40°C	TAS^c^
Optimum temperature	30–45°C	TAS^c^
pH	8.0–10.0	TAS^c^
Carbon source	Phototroph, mixotroph	TAS^c^
Energy source	Phototroph	TAS^c^
Relationship to oxygen	Aerobic	TAS^c^
Pathogenicity	None	NAS
Origin	Natron Lake, Chad	NAS
Habitat	Freshwater	NAS
Latitude	14.306969 N	NAS
Longitude	18.581542 E	NAS
Obtained from	University of Texas, Strain UTEX 2340	NAS

*Evidence codes: IDA, Inferred from Direct Assay (first time in publication); TAS, Traceable Author Statement. TAS^a^ : (Muhling, 2000), TAS^b^ : (Furmaniak et al., 2017), TAS^c^ : (Vonshak, 1997); NAS, Non-traceable Author Statement. These evidence codes are from the Gene ontology Project (Ashburner et al., 2000).*

**TABLE 2:** Genome assembly details and summary statistics for *Limnospira fusiformis* SAG 85.79 (MIGS standard, Field et al., 2008).

Project summary		
Genome assembly details		
Property		Term
Finishing quality		Complete
Libraries used		Nanopore 1D genomic DNA by Ligation SQK-LSK109
Sequencing platforms		Oxford Nanopore MinION, Illumina MiSeq
Fold coverage		114.0×
Assemblers		Shasta v. 0.4.0
Gene calling method		Prokaryotic Genome Annotation Pipeline (https://github.com/ncbi/pgap)
Genome database release		Genbank
Genbank ID		NZ_CP051185.1
Project relevance		Biotechnology

**Genome statistics**		

**Property**	**Term**	**% total**

Genome size (bp)	6,423,694	100.00%
DNA coding region (bp)	5,317,886	82.8%
DNA G+C content (bp)	2,882,690	44.9%
Total genes	5,994	100%
Protein coding genes	5,344	89.2%
RNA genes	51	0.85%
rRNA operons	2	
tRNAs	41	
CRISPR arrays	7	

Genome completeness is an important measure for assembly quality. One method to assess genome completeness is to identify core genes of the clade. We used BUSCO to assess genome completeness by tabulating the 773 single-copy core cyanobacterial orthologs (Seppey et al., 2019). The BUSCO score was 98.9% (765/773 complete). Of these complete genes, five were duplicated. Additionally, there were three genes that were fragmented and five that were reported missing. With further analysis of the five missing genes using NCBI tblastn and blastp (Altschul et al., 1990): two were present in alternate forms (inositol monophosphatase and n-acetyltransferase); recF and riboflavin synthase subunit alpha had 3′ truncations possibly derived from sequencing errors; and like all other *Limnospira* species, miniribonuclease 3 is also not present in *L. fusiformis* but ribonuclease 3 is present.

The genomes of *L. fusiformis* and its close relative *Arthrospira* sp. TJSD092 are within 10.7 kb in size and share the same 44.9% GC content (GCA_003060805.1). The DNA coding region (CDS) comprises 82.8% of the genome, with a total of 5,994 genes, 5,344 of which are protein-coding and 51 are RNA genes. The genome contains two rRNA operons and 41 tRNAs.

Selfish DNA elements tend to be repetitive, and we found that 29.6% of the genome is annotated as repetitive DNA (Geneious v11.0.5). Selfish DNA elements were abundant in the *L. fusiformis* genome and included 518 transposases, 12 recombinases, 89 toxin/antitoxin elements, and seven CRISPR arrays. We also found 37 reverse transcriptases and eight phage annotations, indicating possible viral associations with this genome. Furthermore, these sequences appeared to be widespread among *Limnospira*, where we found homologues of these genes using blastx (E < e-36, ≥ 98% coverage, data not shown), suggesting that these selfish elements are endemic to the clade.

*L. fusiformis* contains the HsdR, HsdM, and HsdS type I restriction modification system as well as a Res-Mod type III system. The seven CRISPR clusters are comparable to the three-to-nine clusters present in other “Spirulina” genomes (Table 3). The genome’s type I and III restriction modification systems and CRISPRs are the main defense mechanisms that inhibit the stable transformation of *Arthrospira* (Cheevadhanarak et al., 2012; Jeamton et al., 2017).

**TABLE 3:** Summary of *Limnospira* and “*Arthrospira*” genome assemblies.

Strain	Assembly level	Sequencing technology	Coverage	N50 (bp)	References
*L. fusiformis* SAG 85.79	Complete	Nanopore; Illumina	114×	6,423,694	This study GCA_012516315.1
*“Arthrospira* sp.*”* TJSD092	Complete	Illumina; PacBio	190×	6,434,389	Genbank accession: GCA_003060805.1
*“Arthrospira* sp.*”* PCC 9108	Complete	PacBio	191×	6,763,964	Genbank accession: GCA_016745315.1
*L. indica* PCC 8005	Chromosome	454; Sanger	n/a	1,412,831	Janssen et al., 2010: GCA_000973065.1
*Limnospira* sp. BM01	Chromosome	Illumina	6.43×	61,454	Genbank accession: GCA_014250495.1
*“A. platensis”* NIES-39	Chromosome	ABI 3730	11×	619,347	Fujisawa et al., 2010: GCA_000210375.1
*“A. platensis”* C1	Chromosome	454; Sanger	28×	206,210	Cheevadhanarak et al., 2012: GCA_000307915.1
*“A. platensis”* YZ	Chromosome	ABI 3730; Illumina	86×	1,054,592	Xu et al., 2016: GCA_001611905.1
*Limnospira fusiformis* KN	Scaffold	Illumina	350×	32,852	Genbank accession: GCA_014489865.1
*“A. maxima” CS-328*	Contig	n/a	n/a	92,573	Carrieri et al., 2011: GCA_000173555.1
*“A. platensis”* NIES-46	Contig	MiSeq	42×	40,752	Suzuki et al., 2019: GCA_009176225.1
*A. platensis* Paraca	Contig	Illumina	36×	72,660	Lefort et al., 2014: GCA_000175415.3
*“Arthrospira* sp.*”* TJSD091	Contig	Illumina	130×	49,578	Dong et al., 2015: GCA_000974245.1
*“A. platensis”* O9.13F	Contig	Illumina	20×	6,514	Genbank accession: GCA_003268325.1
*“A. platensis”* FACHB-971	Contig	Illumina	50×	39,263	Genbank accession: GCA_014698385.1
*“A. platensis”* FACHB-439	Contig	Illumina	50×	44,370	Genbank accession: GCA_014698675.1
*“A. platensis”* FACHB-835	Contig	Illumina	50×	34,814	Genbank accession: GCA_014698815.1
*Arthrospira* sp. PLM2.Bin9	Contig	Illumina	18×	36,014	Genbank accession: GCA_007732545.1

*Assembly level as described previously (Kitts et al., 2016).*

#### Phylogenetic Analysis Confirmed *L. fusiformis* Placement in the *Limnospira* Clade

**FIGURE 3:**
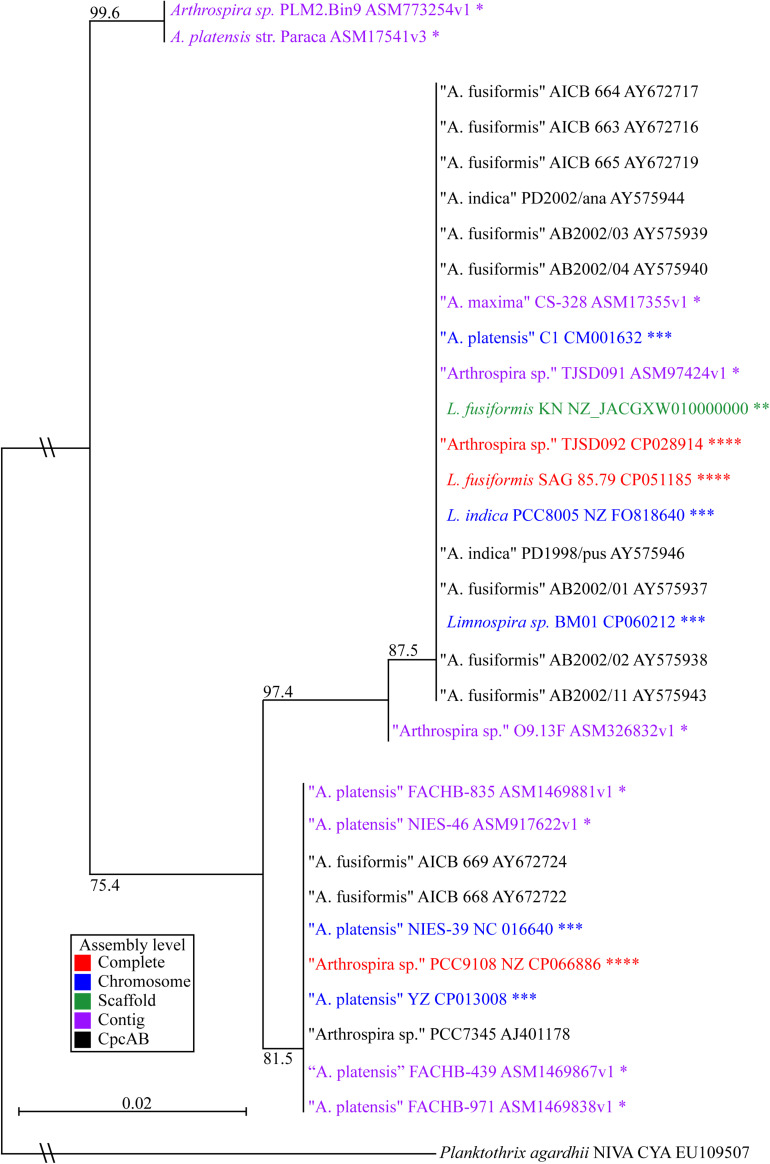
*Limnospira* phylogenetic tree based on *cpcAB* loci. The sequence sources of the *cpcAB* loci are denoted: complete genome (red ^****^), chromosome assembly (blue ^∗∗∗^), scaffold assembly (green ^∗∗^), contig assembly (purple ^∗^), *cpcAB* sequence only (black). Bootstrap values of major branch points are shown. Quotation marks are used to indicate species proposed for *Limnospira* membership.

We built a phylogenetic tree based on the phycocyanin alpha and beta subunits, *cpcAB* (Figure 3; Manen and Falquet, 2002). The tree includes all *Arthrospira* and *Limnospira* genome assemblies, as well as additional strains that were previously placed in the *Limnospira* clade with publicly available *cpcAB* sequences (Nowicka-Krawczyk et al., 2019).

Figure 3 shows *L. fusiformis* and most of the “*Arthrospira”* strains placed in the *Limnospira* clade (Nowicka-Krawczyk et al., 2019). *A. platensis* Paraca and *Arthrospira* sp. PLM2.Bin9 are the only strains not clearly in the *Limnospira* clade. Consistent with previous results (Cheevadhanarak et al., 2012; Nowicka-Krawczyk et al., 2019), the *Limnospira* clade is split into two major groups (Figure 3). Of the sequenced genomes (Table 3), *Arthrospira* sp. PCC 9108 and *A. platensis* NIES-39, NIES-46, YZ, FACHB-971, FACHB-439, and FACHB-835 are grouped together in one branch while a second group contains *L. fusiformis* SAG 85.79, *Arthrospira* sp. TSJD092, *Limnospira* sp. BM01, *L. fusiformis* KN, *Arthrospira* sp. TSJD091, *A. platensis* C1, *A. maxima* CS-328, *L. indica* PCC 8005, and *Arthrospira* sp. O9.13F (Figure 3).

#### Extrachromosomal Elements Were Not Detected

A report from 1993 (Song et al., 1993) indicated the presence of a plasmid in *Spirulina platensis*, however, this finding has not been described elsewhere (Fujisawa et al., 2010; Cheevadhanarak et al., 2012; Lefort et al., 2014). Therefore, we conducted a search for circular extrachromosomal DNA elements within the *L. fusiformis* genome. We used PlasmidSPAdes and Bandage to identify circular contigs within the Illumina library and screened the elements that were present in both outputs (Wick et al., 2015; Antipov et al., 2016). Our search for extrachromosomal elements was conducted with the short-read axenic library in order to exclude contaminating elements present in the long-read xenic library. We did not identify any extrachromosomal elements belonging to *L. fusiformis* (the PhiX Control v3 circular spike-in Illumina control library was present, as expected).

### Axenicity

#### Selective Gas Vesicle Collapse Is an Effective Physical Treatment

Gas vesicles allow *A. platensis* cells to float during log-phase (Kim et al., 2005). If these vesicles proved to be more robust than the vesicles of contaminating species, this property could be used as a separation method. Under phase-contrast microscopy, gas vesicles appear as bright irregular shapes within these ∼80 μm buoyant trichomes (Cohen-Bazire et al., 1969; Sili et al., 2012). Gas vesicles collapse under pressure, causing cells to lose their buoyancy and appear darker and more hollow (Cohen-Bazire et al., 1969; Walsby and Bleything, 1988). We cultured xenic *L. fusiformis* under standard growth conditions and observed buoyancy from day three to at least day eight. These xenic cultures were pressurized with centrifugal forces at 2,000, 4,000, 8,000, and 16,000 g, and selective separation was found at 8,000 g (Supplementary Figures 3–11).

We enriched for *L. fusiformis* using selective gas vesicle collapse to deplete contaminating microbes. *L. fusiformis* cells were briefly vortexed to remove contaminating bacteria attached to the trichomes (Figure 2A), filtered at 40 μm, centrifuged to pellet the contaminants (Figure 2C), then transferred from the surface layer to sterile media (Figure 2B). Dark-field microscopy images at each stage confirmed the purification (Figures 2A–C and Supplementary Figures 12–14). We implemented these findings as a novel axenicity technique—selective gas vesicle collapse.

#### An Axenic *L. fusiformis* Culture Was Established Under Antibiotic and Alkaline Conditions

*L. fusiformis* tolerates elevated alkaline conditions and specific antibiotics, unlike microcystin-producing species (i.e., *Microcystis aeruginosa, Oscillatoria* spp., and *Nostoc* spp.), which often contaminate “Spirulina” supplements (Moreno et al., 2004; Sena et al., 2011; Roy-Lachapelle et al., 2017; Borowitzka, 2018; Khong et al., 2019). We found the highest tolerable pH (12.15) also caused contaminating species (i.e., *Microcystis* spp.) to lyse (Figures 2D,E and Supplementary Figures 15, 16). This high pH combined with an antibiotic cocktail selected against any residual contaminating species while minimizing damage to *L. fusiformis*, resulting in an axenic culture (Figure 2F and Supplementary Figure 17; Choi et al., 2008; Sena et al., 2011).

#### Microscopy and rDNA Sequence Analysis Support Axenicity of Prepared Culture

The treated culture was analyzed under dark-field microscopy and no contaminating organisms were detected. We then compared sequencing data from samples that had only undergone physical treatments—represented by the nanopore data—with samples that were subjected to physical and chemical treatments—represented by the Illumina data. We conducted a computational search for 16s and 23s ribosomal RNA gene sequences within these respective data sets. Four contaminating ribosomal gene sequences were identified within the nanopore data, with best matches to: *Coraliomargarita akajimensis, Lishizhenia caseinilytica, Halomonas desiderata*, and a member of *Xanthomonadacae*. When physical and chemical treatments were combined (see section “Materials and Methods” for details), only ribosomal RNA gene sequences from *L. fusiformis* were detected. Based on these results we concluded that physical treatment alone was not sufficient to induce axenicity. However, axenicity was achieved when physical treatment was used in combination with chemical treatment.

## Discussion

Long-read sequencing enabled closure of the *Limnospira fusiformis* genome in a single pass. The 6.42 Mb genome, assembled as a single circular chromosome, required the scaffolding power of long reads (>35 kb) and the accuracy afforded by the short-read library. Assemblies of this highly repetitive “Spirulina” family have been previously attempted without the guidance of long-reads; multi-contig drafts or incorrect genome structure can be the result (Alkan et al., 2011; Tørresen et al., 2019). Indeed, the only closed genomes in the group have been possible with long-read produced scaffolds (Table 3). We believe our approach allows for a simple assembly that is less error prone when compared to using short-read data alone.

Nowicka-Krawczyk proposed the *Limnospira* clade using rRNA nucleotide sequences of 64 “*Arthrospira”* strains and established *L. fusiformis*, *L. indica*, and *L. maxima* as the founding members of the genus (Nowicka-Krawczyk et al., 2019). Phylogenetic analysis using published *cpcAB* sequences (Manen and Falquet, 2002) from that clade combined with the currently available *Arthrospira* genomes (Table 3) shows that most of these are grouped with *Limnospira*, with the exception of *A. platensis* Paraca and *Arthrospira* sp. PLM2.Bin9 (Figure 3). Furthermore, the optimal pH conditions derived from publications, media recipes, or the isolate geography establishes the *Limnospira* clade with an alkaline preference (pH 8–10), as described by Nowicka-Krawczyk (ATCC medium 1679); (BioSample: SAMN10237416; Vonshak, 1997; Şeker et al., 2008; Carrieri et al., 2011; Cheevadhanarak et al., 2012; Miklaszewska et al., 2012; Lefort et al., 2014; Dong et al., 2015; Shiraishi, 2015; Dineshkumar et al., 2016; Xu et al., 2016; Suzuki et al., 2019; Yadav et al., 2020; Zhao et al., 2020). With *A. platensis* Paraca and *Arthrospira* sp. PLM2.Bin9 as an outgroup, we propose that the two subclades (Figure 3) should be reclassified as *Limnospira*, which is consistent with Nowicka-Krawczyk’s rRNA analysis.

In contrast with previous studies which provided time-intensive broad-spectrum or rapid, contaminant-specific approaches, we focused on rapid and broad-spectrum selection using sequencing to verify axenicity (Table 4). Broad-spectrum selection is important when contaminating species are unknown. The protocol we have developed can be completed in 7 days, and the use of antibiotics, elevated pH, and selective gas vesicle collapse provides the necessary selection.

**TABLE 4:** “*Arthrospira*” and *Limnospira* axenicity methods.

Method	Description	Time required	Verification	Strain
Shiraishi (2015)	Washing by vortexing and filtration Individual trichomes selected for axenic culture propagation	>1 month	Microscopy and agar plating	*A. platensis UTEX* 1926
Sena et al. (2011)	Washing by filtration pH treatment Antibiotic treatment Propagation Serial dilutions	∼3 weeks	Microscopy and agar plating	*A. platensis* Lefevre 1963/M-132-1
Choi et al. (2008)	Washing by centrifugation Antibiotic treatment	7 days	Microscopy and agar plating	*A. platensis* SAG 21.99
Physical and chemical treatments (this work)	Surface layer collection from culture Washing by vortexing and filtration Washing by centrifugation and surface layer extraction Antibiotic and pH treatment	7 days	Microscopy and 16s and 23s rDNA identification	*L. fusiformis* (formerly known as *A. platensis* SAG 85.79)

We have provided an accurate reference genome and a rapid axenicity method for *Limnospira fusiformis* and now propose the optimization of genetic engineering methods as the next step in progressing *L. fusiformis* research. Two stable transformation methods are described for *Limnospira* species—*Rhizobium radiobacter* (syn. *Agrobacterium tumefaciens*)-based DNA transfer and Tn5 transposon-mediated genome manipulation (Jeamton et al., 2017; Dehghani et al., 2018). Both methods integrate DNA into non-specific genomic loci but targeted edits may be possible when used in conjunction with other molecular tools (Vergunst et al., 1998; Tzfira et al., 2003; Fan et al., 2015; Chen and Wang, 2019). Targeted mutations to the restriction modification system in *L. fusiformis* would be an ideal target for improving its transformation efficiency, as has been done in other organisms (Hoshino et al., 1980; Kretz et al., 1991; Hobson et al., 2008; Ferri et al., 2010). With the rapid proliferation of genetic engineering tools, new methods for customizing *L. fusiformis* will become applicable as it finds continued success in the global market.

## Data Availability Statement

The datasets presented in this study can be found in online repositories. The names of the repository/repositories and accession number(s) can be found below: https://www.ncbi.nlm.nih.gov/, PRJNA623410.

## Author Contributions

MH, LM, and DB devised the project and designed the experiments. DB and LM supervised the project. MH performed the genome experiments. MH and T-KT-D performed the axenicity experiments. All authors contributed to bioinformatic analysis, wrote, and approved the manuscript.

## Conflict of Interest

LM has received reimbursement for travel, accommodation and conference fees to speak at events organized by ONT. The authors declare that this study received materials from Zymo Research, Biomatters Inc., and Avantor Biosciences. These sponsors were not involved in the study design, collection, analysis, interpretation of data, the writing of this article or the decision to submit it for publication.

## Footnotes

1 https://github.com/nanoporetech/bonito

2 https://github.com/kishwarshafin/pepper

3 https://github.com/ncbi/pgap

4 https://github.com/jstjohn/SeqPrep
