# Supplementary material for: De-novo Assembly of Limnospira fusiformis Using Ultra-Long Reads
Source: Front Microbiol. 2021 Apr 16;12:657995. doi: 10.3389/fmicb.2021.657995 (PMC8085491; doi:10.3389/fmicb.2021.657995)
Supplement: Supplementary file 1 [file Data_Sheet_1.PDF]

Supplementary Material

1 Supplementary Figures and Tables

1.1 Supplementary Figures

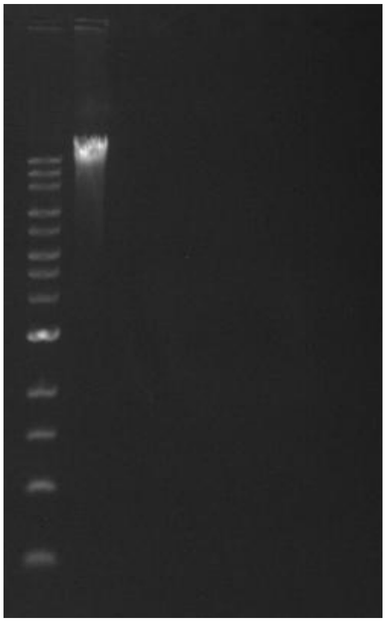

Supplementary Figure 1. 0.5 µg of DNA extracted from the physically treated *L. fusiformis* visualized on a 0.5% agarose gel alongside a NEB 1 kb extend ladder (N3239S), the largest band of which is 48.5 kb. This DNA was size selected and used for Nanopore sequencing.

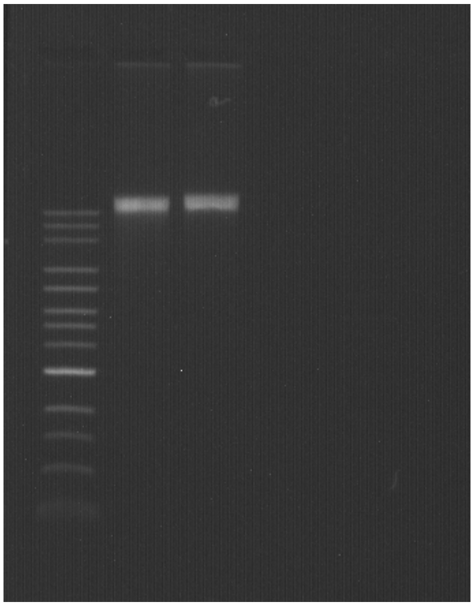

Supplementary Figure 2. 0.5 µg of DNA extracted from the axenic *L. fusiformis* visualized on a 0.5% agarose gel alongside a NEB 1 kb extend ladder (N3239S), the largest band of which is 48.5 kb. Lane 2 is from the main DNA extraction, and lane 3 is from a back-extraction.

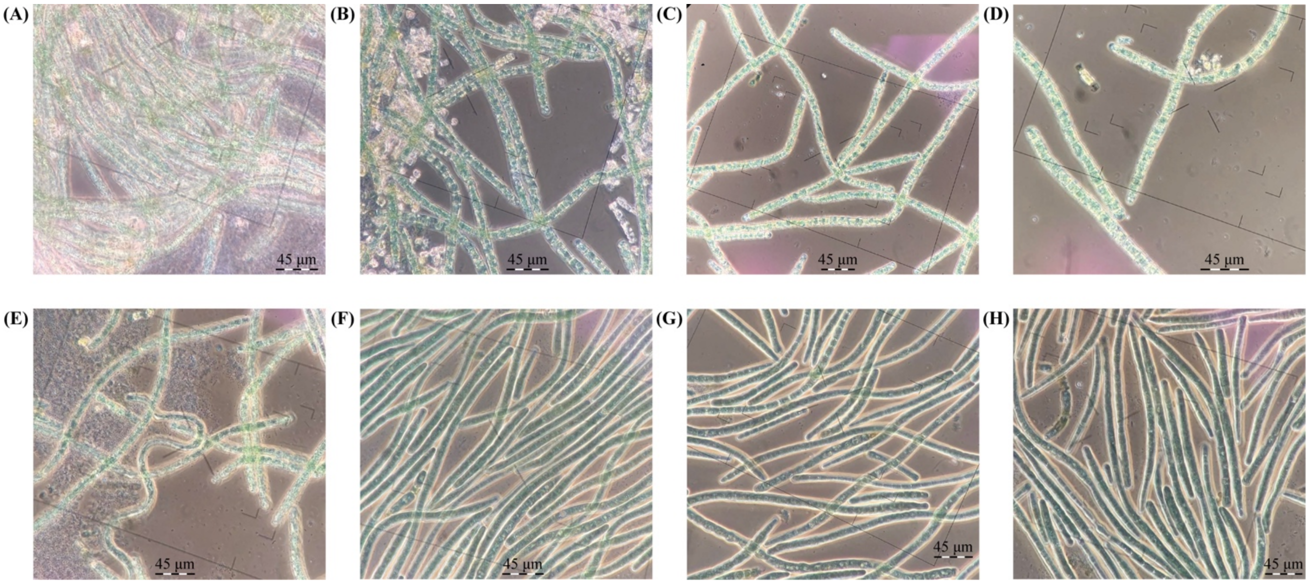

Supplementary Figure 3. (**A-D**) Floating *L. fusiformis* cells after centrifugation at (**A**) 2000 g, (**B**) 4000 g, (**C**) 8000 g, (**D**) 16000 g. (**E-H**) Pelleted *L. fusiformis* cells after centrifugation at (**E**) 2000 g, (**F**) 4000 g (**G**) 8000 g, (**H**) 16000 g.

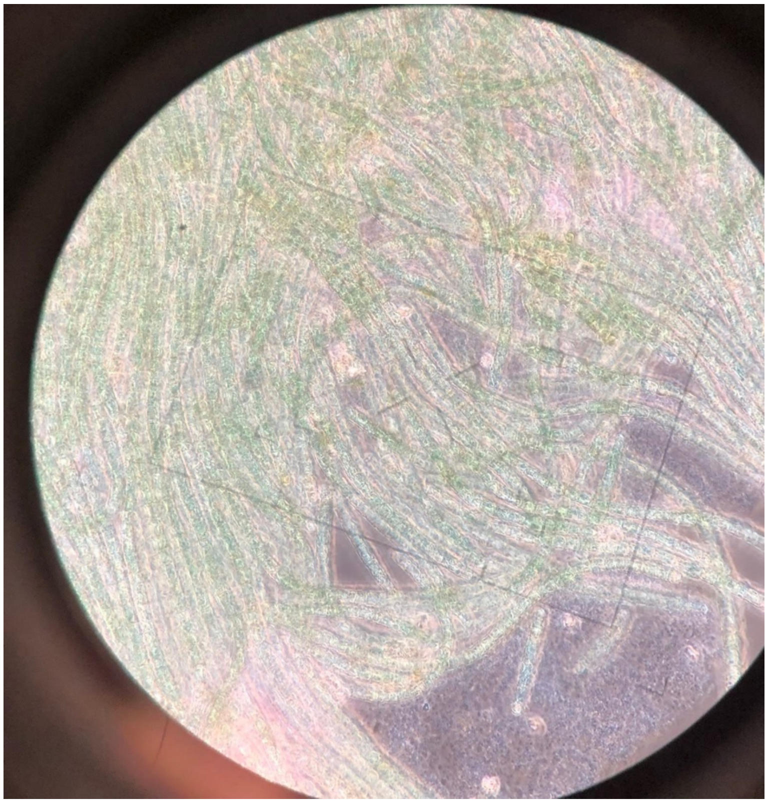

Supplementary Figure 4. Uncropped phase contrast image of Figure S3A.

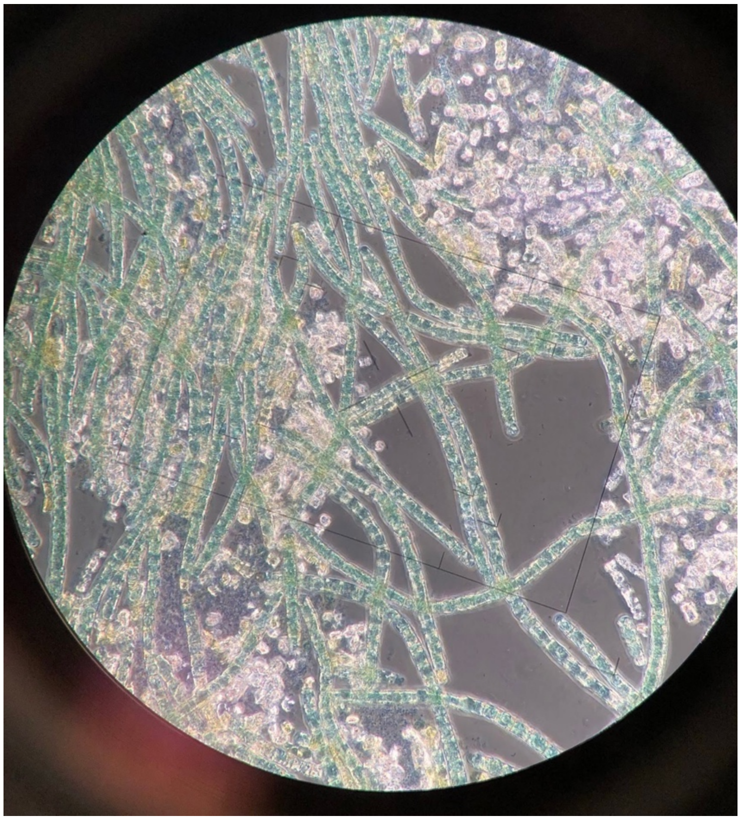

Supplementary Figure 5. Uncropped phase contrast image of Figure S3B.

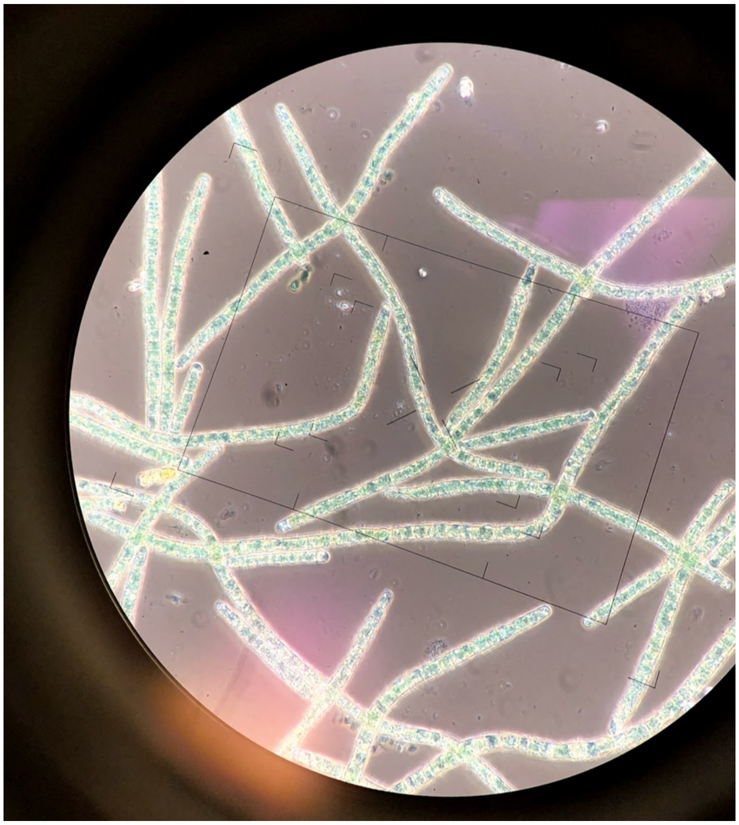

Supplementary Figure 6. Uncropped phase contrast image of Figure S3C.

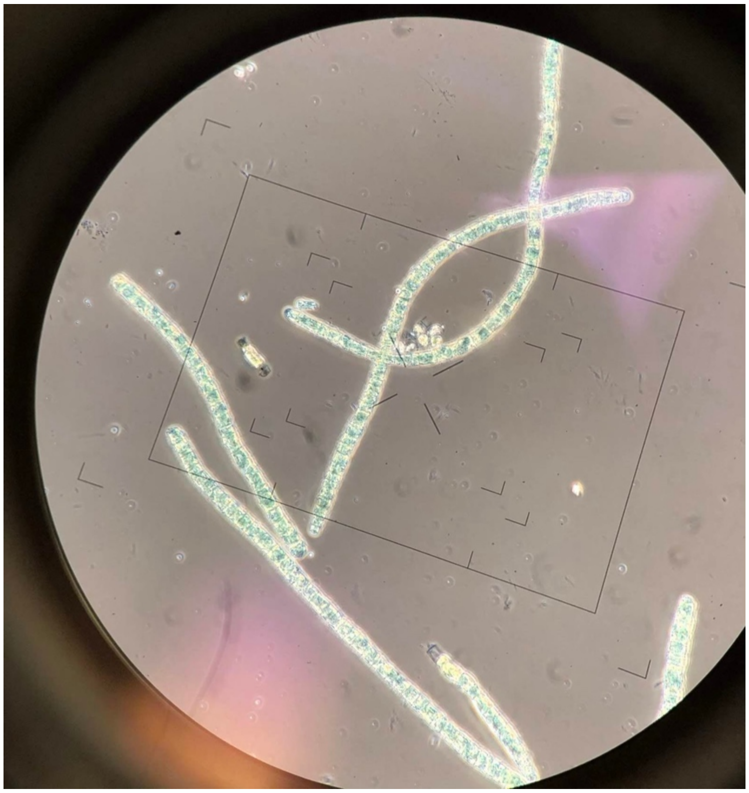

Supplementary Figure 7. Uncropped phase contrast image of Figure S3D.

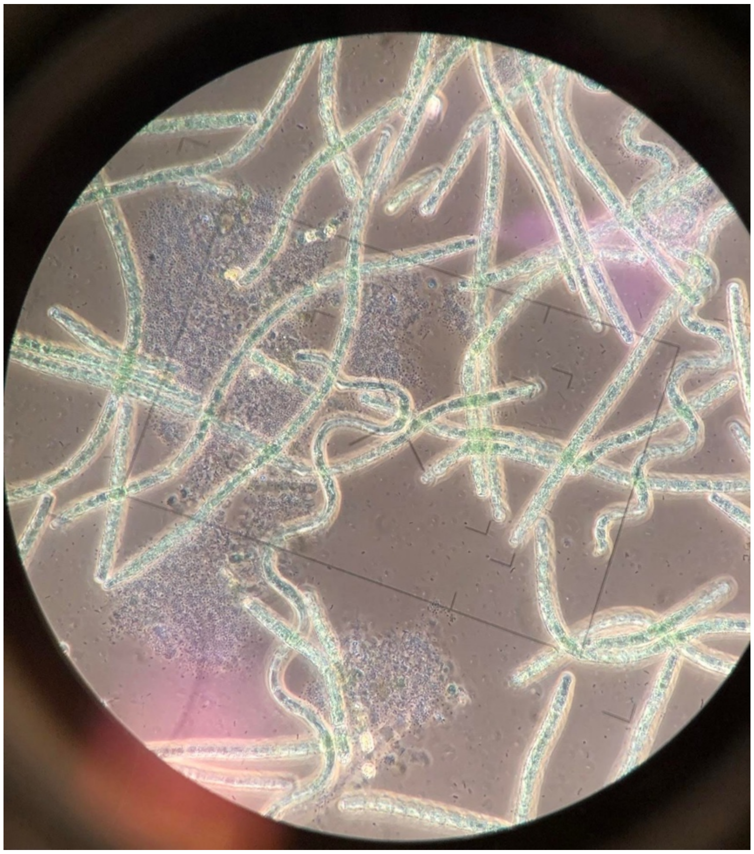

Supplementary Figure 8. Uncropped phase contrast image of Figure S3E.

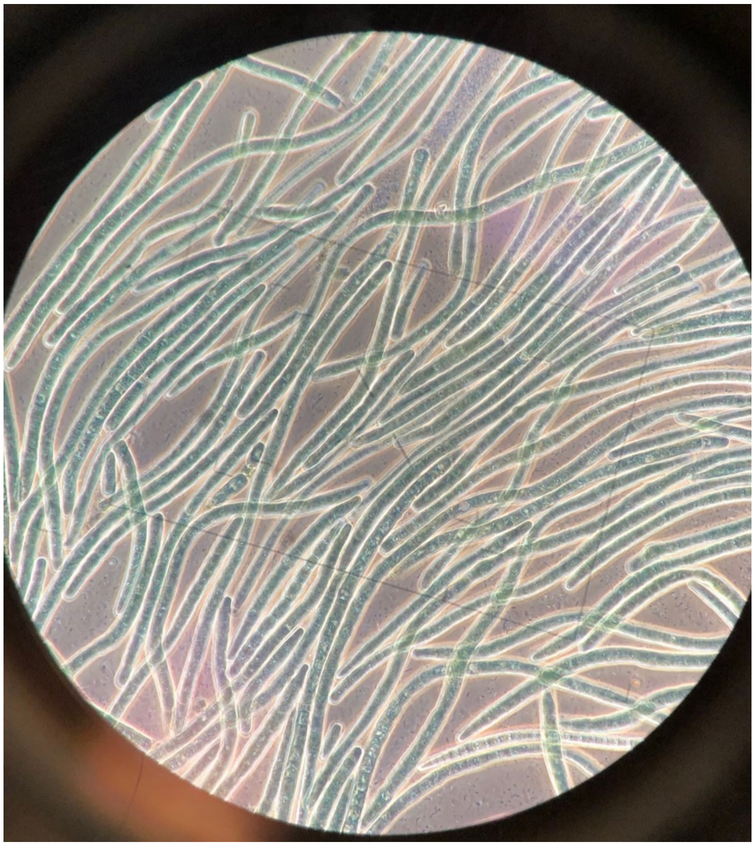

Supplementary Figure 9. Uncropped phase contrast image of Figure S3F.

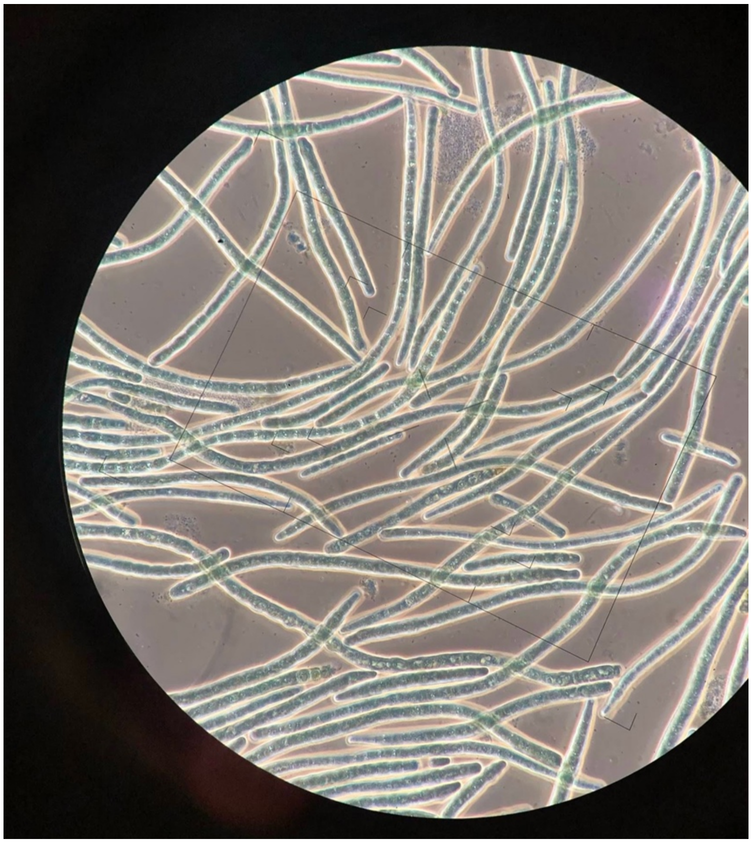

Supplementary Figure 10. Uncropped phase contrast image of Figure S3G.

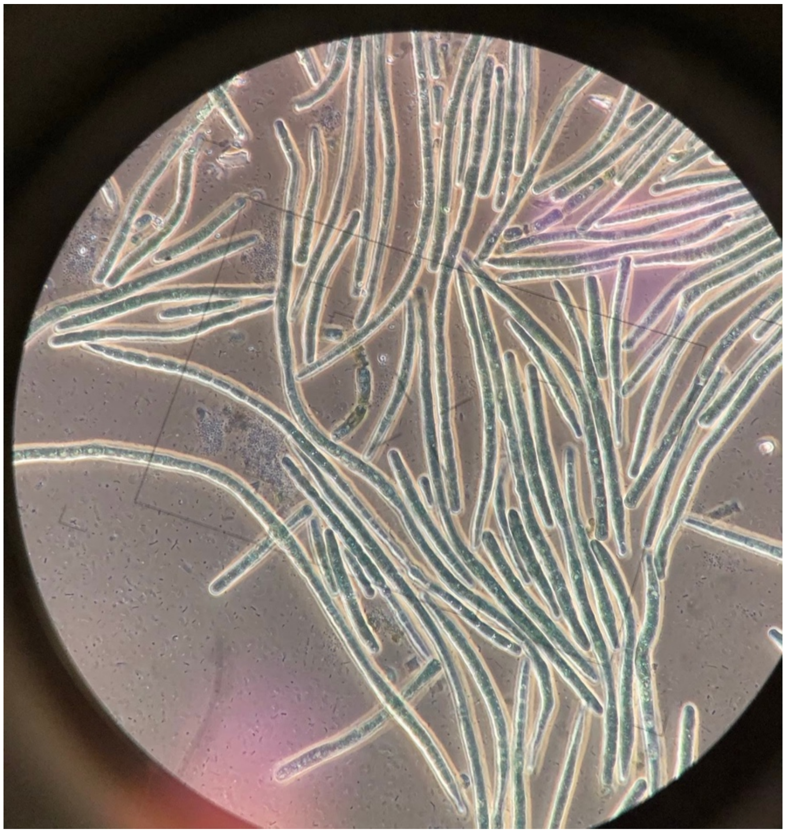

Supplementary Figure 11. Uncropped phase contrast image of Figure S3H.

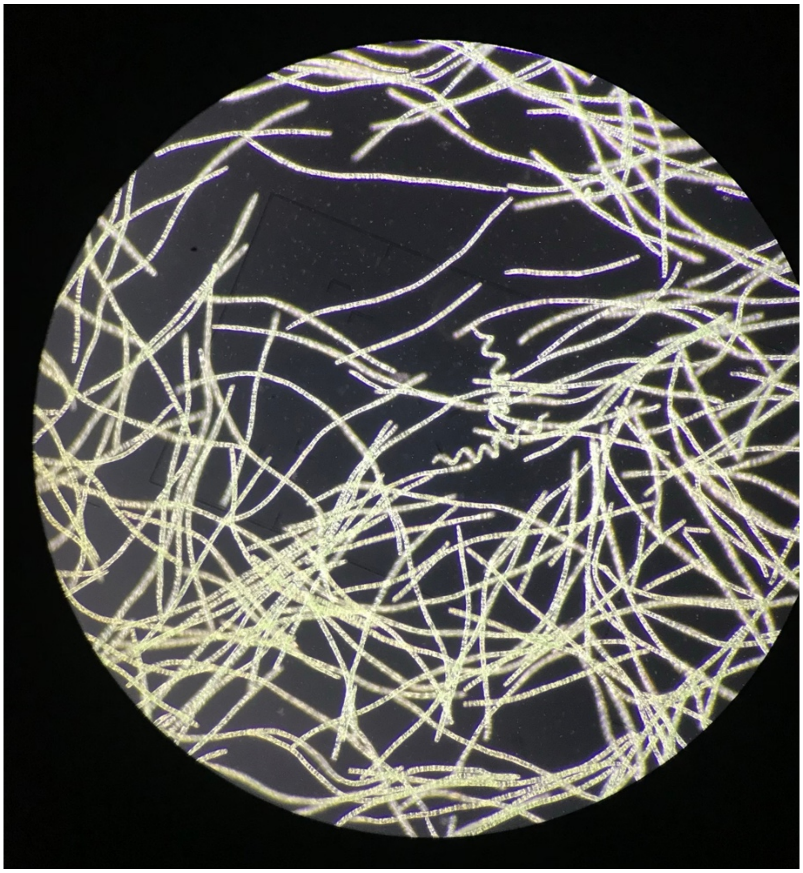

Supplementary Figure 12. Uncropped dark field image from Figure 3A.

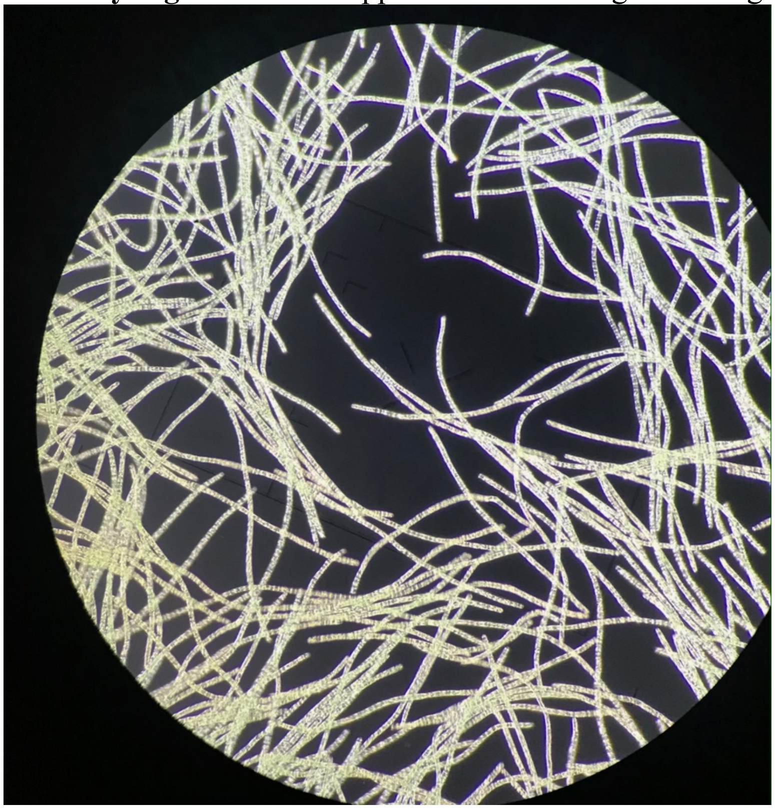

Supplementary Figure 13. Uncropped dark field image from Figure 3B.

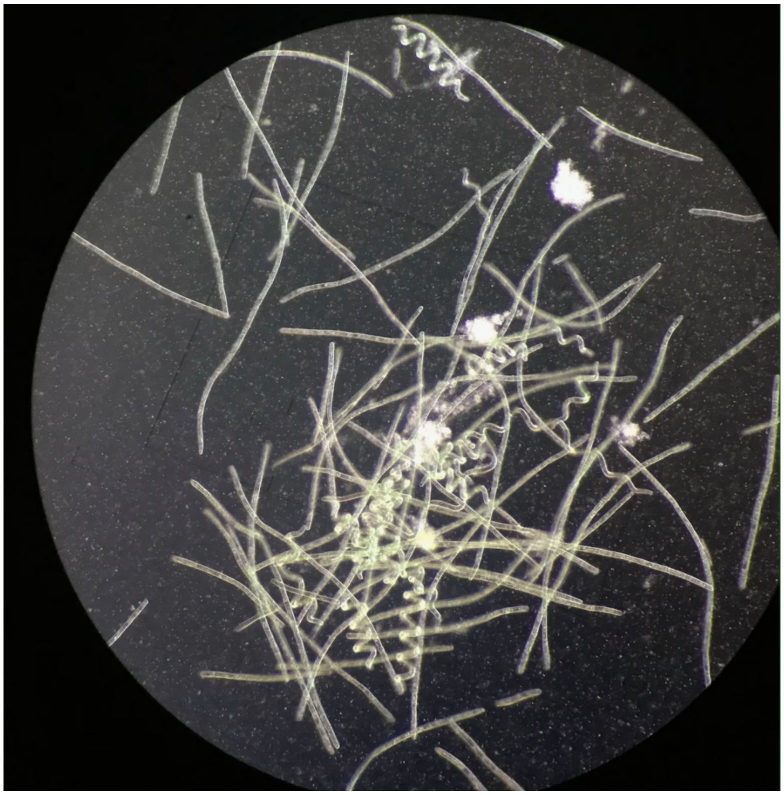

Supplementary Figure 14. Uncropped dark field image used for Figure 3C.

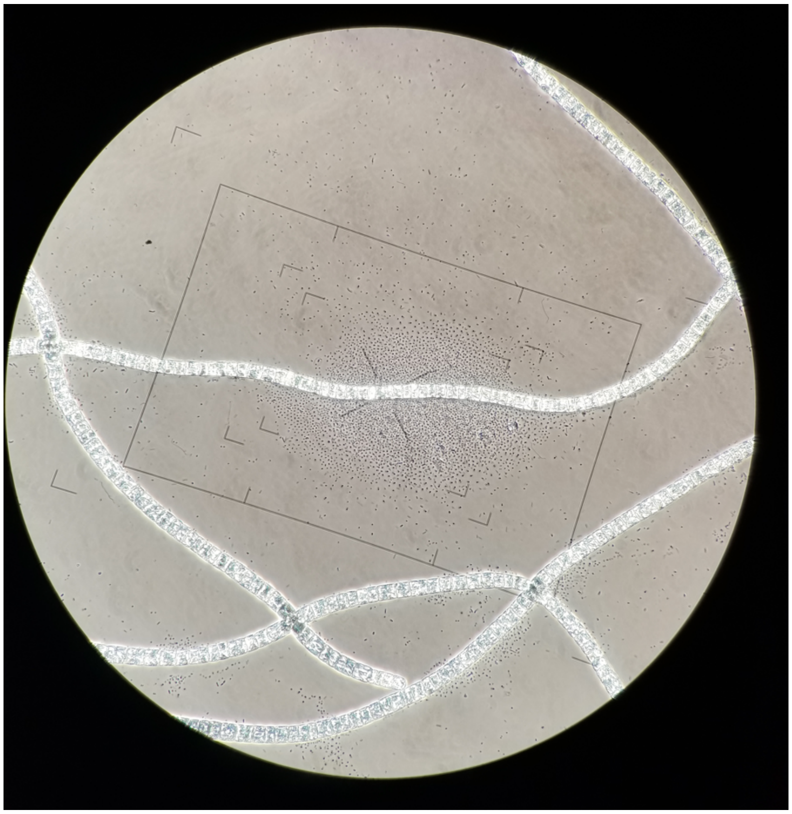

Supplementary Figure 15. Uncropped phase contrast image used for Figure 3D.

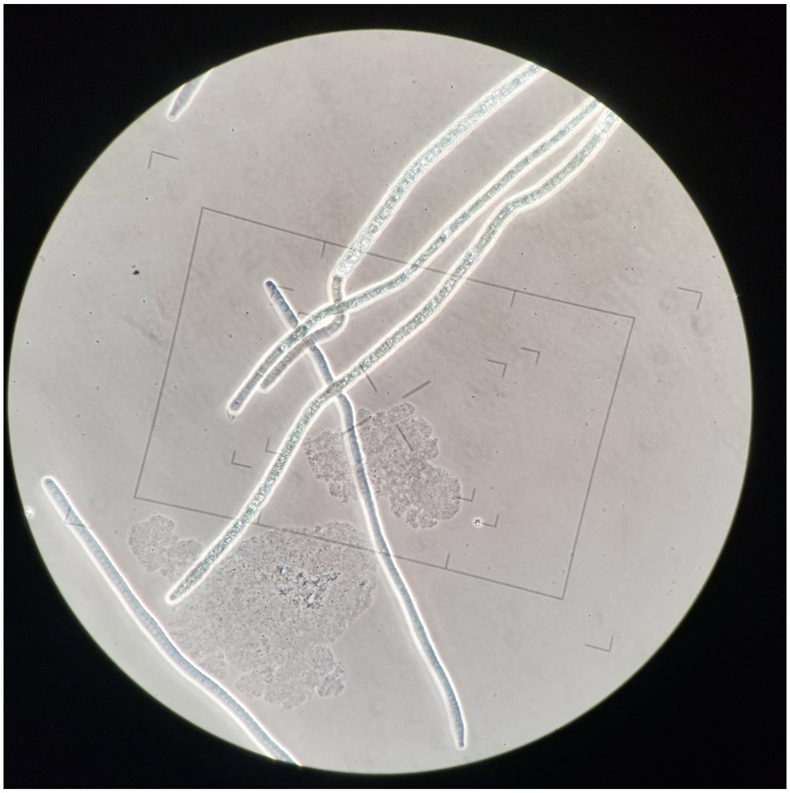

Supplementary Figure 16. Uncropped phase contrast image used for Figure 3E.

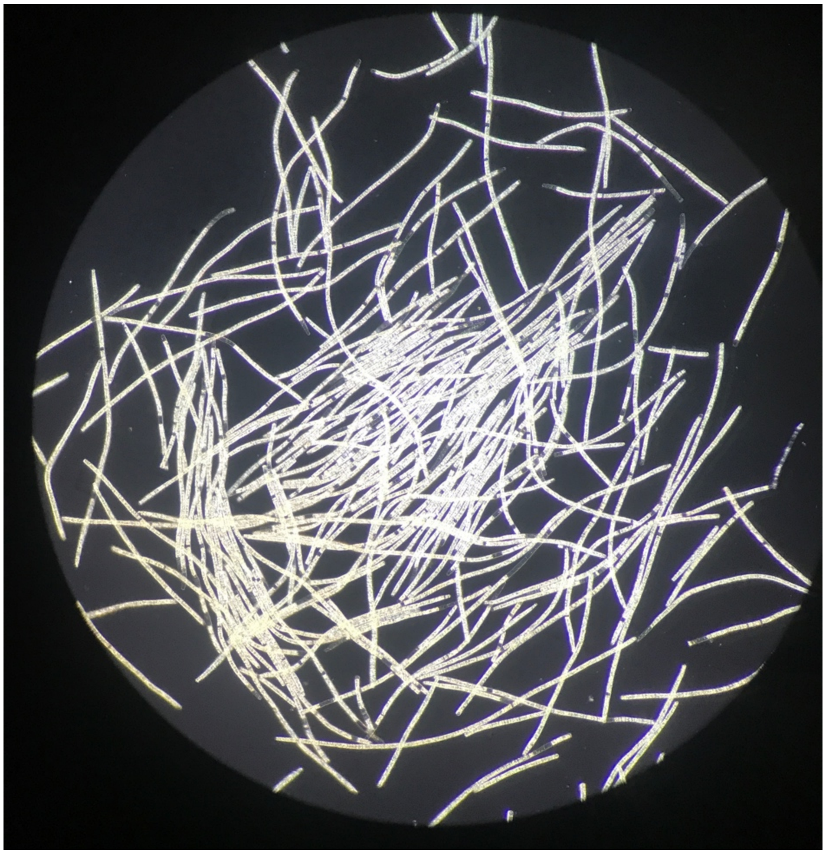

Supplementary Figure 17. Uncropped dark field image used for Figure 3F.
